# Genetic overlap and causality between COVID-19 and multi-site chronic pain: the importance of immunity

**DOI:** 10.3389/fimmu.2024.1277720

**Published:** 2024-03-18

**Authors:** Yanjing Chen, Ping Liu, Zhiyi Zhang, Yingling Ye, Sijie Yi, Chunhua Fan, Wei Zhao, Jun Liu

**Affiliations:** ^1^ Department of Radiology, Second Xiangya Hospital, Central South University, Changsha, China; ^2^ Fujian University of Traditional Chinese Medicine, Fuzhou, China; ^3^ Clinical Research Center for Medical Imaging in Hunan Province, Changsha, Hunan, China

**Keywords:** GWAS, COVID-19, genetic overlap, pleiotropy, chronic multi-site pain, inflammation

## Abstract

**Background:**

The existence of chronic pain increases susceptibility to virus and is now widely acknowledged as a prominent feature recognized as a major manifestation of long-term coronavirus disease 2019 (COVID-19) infection. Given the ongoing COVID-19 pandemic, it is imperative to explore the genetic associations between chronic pain and predisposition to COVID-19.

**Methods:**

We conducted genetic analysis at the single nucleotide polymorphism (SNP), gene, and molecular levels using summary statistics of genome-wide association study (GWAS) and analyzed the drug targets by summary data-based Mendelian randomization analysis (SMR) to alleviate the multi-site chronic pain in COVID-19. Additionally, we performed a latent causal variable (LCV) method to investigate the causal relationship between chronic pain and susceptibility to COVID-19.

**Results:**

The cross-trait meta-analysis identified 19 significant SNPs shared between COVID-19 and chronic pain. Coloc analysis indicated that the posterior probability of association (PPH4) for three loci was above 70% in both critical COVID-19 and COVID-19, with the corresponding top three SNPs being rs13135092, rs7588831, and rs13135092. A total of 482 significant overlapped genes were detected from MAGMA and CPASSOC results. Additionally, the gene ANAPC4 was identified as a potential drug target for treating chronic pain (P=7.66E-05) in COVID-19 (P=8.23E-03). Tissue enrichment analysis highlighted that the amygdala (P=7.81E-04) and prefrontal cortex (P=8.19E-05) as pivotal in regulating chronic pain of critical COVID-19. KEGG pathway enrichment further revealed the enrichment of pleiotropic genes in both COVID-19 (P=3.20E-03,Padjust=4.77E-02,hsa05171) and neurotrophic pathways (P=9.03E-04,Padjust =2.55E-02,hsa04621). Finally, the latent causal variable (LCV) model was applied to find the genetic component of critical COVID-19 was causal for multi-site chronic pain (P=0.015), with a genetic causality proportion (GCP) of was 0.60.

**Conclusions:**

In this study, we identified several functional genes and underscored the pivotal role of the inflammatory system in the correlation between the paired traits. Notably, heat shock proteins emerged as potential objective biomarkers for chronic pain symptoms in individuals with COVID-19. Additionally, the ubiquitin system might play a role in mediating the impact of COVID-19 on chronic pain. These findings contribute to a more comprehensive understanding of the pleiotropy between COVID-19 and chronic pain, offering insights for therapeutic trials.

## 1 Introduction

COVID-19 is an infectious respiratory tract disease caused by the severe acute respiratory syndrome coronavirus 2 (SARS-CoV-2). It is estimated that more than 650 million people have been infected with SARS-CoV-2 by the end of 2022 (1). Chronic pain is defined as pain lasting for at least 3 months in one or more systems which has long been a personal and social burden globally. Individuals with chronic pain often report pain at multiple locations, and the number of pain sites is negatively correlated with physical and psychological health. After being infected with the novel coronavirus, the incidence of pain in different parts of the body varies. Muscle pain, headache, and back pain are the most common pain symptoms associated with COVID-19 (2).

Viral infections, including COVID-19, have been associated with bodily pain. This is because the release of certain molecules called cytokines by immune cells and non-neuronal cells, which can directly affect neurons transmitting pain signals, rendering them more sensitive. For instance, in the central nervous system, microglia and astrocyte can release TNF to modulate pain signals within CNS neural circuits (3). Animal experiments have demonstrated that cytokine IL-10 can mediate the downregulation of voltage-gated sodium channel expression in nociceptors (4). A meta-analysis estimated that approximately 8-15% of individuals experience headaches six months after recovering from COVID-19 (5). The SARS-CoV-2 virus can bind to ACE2 receptors present in multiple organs, leading to multi-organ damage, including the development of chronic pain (6) and its activation of inflammatory reactions, involving both innate and acquired immune responses, may also contribute to the progression of chronic pain. Additionally, chronic pain, acting as a stressor, often coexists with psychiatric disorders, particularly major depression (7) and insomnia (8), thereby adding complexity to the management of COVID-19 patients. Research has indicated that one in six COVID-19 survivors develops chronic pain after experiencing new-onset pain (9). Several additional receptors also play crucial roles in the infection and pathogenicity of the SARS-CoV-2 virus, contributing to the generation and transmission of pain during the disease process. Neuropilin-1(NPR-1) and transmembrane serine protease receptor 2 (TMPRSS2) have been identified in brain pericytes and astrocytes (10). NPR-1 facilitates virus entry into cells and enhances viral infectivity by promoting the interaction between the virus and ACE2 (11). Furthermore, NPR-1 is implicated in pain signal transduction through the VEGF-A-NRP-1 pathway (12). TMPRSS2 is involved in activating the Spike (S) protein of the SARS-CoV-2 virus, enabling virus-host membrane fusion and facilitating system infection (13). Studies suggests that TMPRSS2 may induce pain through a PAR2-dependent manner (14).

In summary, persistent pain has become a key symptom of long-term COVID-19.

COVID-19 infection can lead to chronic pain, but it is currently unclear whether chronic pain can cause more severe COVID-19 infection or adverse outcomes. Chronic pain is believed to be associated with neuropathic inflammation mediated by multiple cellular cytokines (15). A cohort study from the UK Biobank revealed a significant association between chronic pain and an elevated propensity for hospitalization among individuals infected with COVID-19 (16). The interaction between pain and neuroimmunity is bidirectional, as immune cells release cytokines, lipids, and growth factors that affect the sensitivity of peripheral nociceptor and central nervous system (CNS) neurons, rendering them susceptible to pain (17). In turn, nociceptor actively release neuropeptides to modulate the activity of innate and adaptive immune cells (18). However, the specific mechanisms of the association between multi-site chronic pain (MCP) and COVID-19 remain unclear.

Additionally, a meta-analysis indicated that muscle pain during the acute phase of infection is more common in non-hospitalized patients, suggesting that pain may not be sufficiently recognized as a significant symptom compared to respiratory distress or fever. Consequently, it leads to an underestimation of its impact on the progression of COVID-19.The nociceptive response elicited by pathogen invasion can function as the host’s primary defense mechanism to alert against the potential invasion of pathogens (19). Therefore, it is still worth investigating whether chronic pain is an independent risk factor for long-term COVID-19 and the potential physiological mechanisms behind it.

Genetic researches have provided valuable insights into the complex physiological mechanisms underlying the interaction between COVID-19 and chronic pain. Existing research has identified genetic variations correlated with susceptibility to and severity of COVID-19, particularly those related to the immune system. In a research conducted on the Sardinian population, three HLA (Human Leukocyte Antigen) haplotype were found to be more prevalent among COVID-19 patients compared to healthy controls (20). Furthermore, several variants in IL6 (such as rs140764737, rs142164099, rs2069849) and IL6R (rs2228144, rs2229237, rs2228145) have been implicated in the pathogenesis of COVID-19 and its complications (21). On the other hand, twin and other genetic studies suggest that the heritability of chronic pain ranges from 30% to 70% (22). Various modifications of spinal cord and neuronal proteins can either aggravate or modulate pain (23), suggesting a role for epigenetic mechanisms in this process. These epigenetic mechanisms likely involve neuroinflammation as a physiological pathway (24). It is probable that common genetic factors are shared between chronic pain and COVID-19.Given the lack of specific treatment options for post-COVID pain, a better understanding of the genetic basis of chronic pain is crucial for the clinical use of candidate gene biomarkers that could inform more targeted treatment approaches.

In this study, we employed large-scale Genome-Wide Association Study (GWAS) summary data to conduct a comprehensive genome-wide pairwise trait pleiotropic analysis between three forms of COVID-19 and multi-site chronic pain. The primary objective was to investigate the genetic factors associated with both COVID-19 and multi-site chronic pain, specifically focusing on the identification of shared variants at the Single Nucleotide Polymorphism (SNP), gene, and biological levels. To explore the causal components in the genetic correlations, we utilized a Latent Causal Variable (LCV) model. Ultimately, at the mRNA level, this study aimed to explore therapeutic targets for disease treatment, providing a new perspective for the advancement of targeted medicines. Overall, by identifying shared variants and potential therapeutic targets, we hope to pave the way for novel treatment strategies and improved patient care.

## 2 Materials and methods

**Figure 1:**
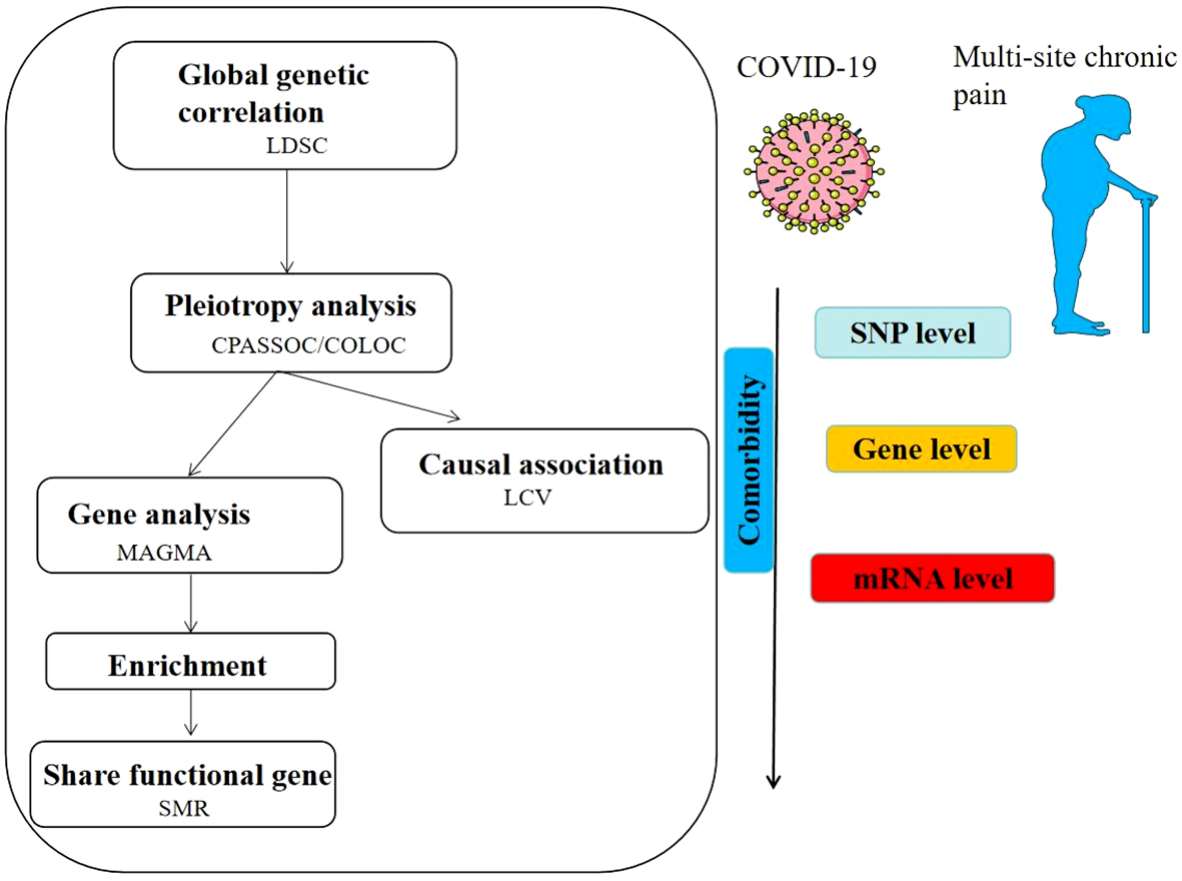
Overview of results of shared genetic architecture between COVID- 19 and multi-site chronic pain.

The flowchart was shown in **Figure 1**. The GWAS for chronic multi-site pain (n=387649) was based on UK Biobank, which is accessible in GWAS catalog (https://www.ebi.ac.uk/gwas/publications/31194737). The COVID-19 Host Genetics Initiative (https://www.covid19hg.org/), is a multinational group that aims to find the genetic variations linked to COVID-19 risk and severity. The COVID-19 HGI GWAS round 7 yielded the COVID-19 data from the European population, which included hospitalized COVID-19, critical COVID-19, and SARS-CoV-2 infection. We first removed SNPs from the data that did not exist in the 1000 Genomes European population in order to standardize the results (25). Next, we eliminated SNPs that had multiple rsIDs or no rsID. Finally, we linked the chromosomal locations of the SNPs to the hg19 reference. The figures were detected from SMART (Servier Medical ART) (genome.https://smart.servier.com/).

### 2.1 General genetic correlation analysis

Linkage disequilibrium score regression (LDSC) enables the estimation of the average sharing of genetic effects across the entire genome between two traits while considering environmental confounders. LDSC software utilizes summary GWAS statistics to estimate heritability (h2) (26) and genetic correlation (rg) (27), ranging from -1 to 1. In this study, rg and h2 between COVID-19 and chronic pain were investigated using the link imbalance score regression software (LDSC, v1.0.1), with reference data obtained from the third phase of the European 1000 Genomes (1KG) project. The LD Score regression intercept was employed to estimate a more powerful and accurate correction factor compared to genomic control.

### 2.2 Cross-trait meta-analysis

Subsequently, we performed a cross-trait meta-analysis using Cross-Phenotype Association (CPASSOC) to identify pleiotropic loci (28). Two statistic method, Shom and Shet are employed in CPASSOC using the summary statistics derived from GWAS to detect cross-phenotype associations. Shom represents an extension of the linear combination of the univariate test statistics allowing for sample size as weights. However, it may be less powerful in the presence of between-study heterogeneity. Therefore, we used SHet to conduct analysis, as it maintains statistical power even when heterogeneity is present by assigning more weights to the larger.

### 2.3 Functional mapping and annotation

The GWAS results often fail to directly identify causal variants due to the influence of linkage imbalances, as well as the non-coding regions or intergenic regions. Furthermore, The Functional mapping and annotation (FUMA) (29) platform provides annotation information for SNPs associated with functional categories. CADD scores above 12.37 indicate potential detrimental effects on protein outcomes. The scores from RegulomeDB offer valuable insight into the regulatory functionality of SNPs by considering their association with expression quantitative trait loci and chromatin marks.

### 2.4 Colocalization analysis

Given that the above analysis relied on a meta-analytic algorithm, the results obtained may encompass genomic regions associated with a single trait. Investigating whether the same genetic variation in loci is responsible for two traits is a more precise way to identify shared genes. The next step involved a colocalization analysis using the coloc package (30), which employs a Bayesian algorithm to calculate posterior probabilities for five exclusive hypotheses related to the sharing of causal variants in a genomic region. These hypotheses include H0 (no association), H1 or H2 (association with one specific trait), H3 (association with both traits, involving two distinct SNPs), and H4 (association with both traits, involving one shared SNP).We extracted summary statistics for variants within 5 Mb of the topSNP at each shared locus detected by FUMA, and a locus was considered colocalized if PPH4 or PPH3 was greater than 0.7.

### 2.5 Genetic level analysis

Due to linkage disequilibrium influence, individual SNP mutations typically do not provide us with effective genetic information. Therefore, in addition to annotating CPASSOC results into genes using FUMA, we also utilize the MAGMA (Multi-marker Analysis of GenoMic Annotation) method (31), which is capable of calculating the joint association of multiple gene sets with the phenotype. After adjustments, we regarded the overlap of genes derived from the MAGMA algorithm and the genes annotated by CPASSOC as more reliable pleiotropic genes.

### 2.6 Enrichment analysis

We conducted an enrichment analysis of the identified pleiotropic genes to better understand the biological implications of these genes (32). Gene Ontology (GO) annotates and classifies the functions of genes and proteins. Kyoto Encyclopedia of Genes and Genomes (KEGG) (33) assigns genes to the functional pathways. GO includes the process of biological processes (BP), molecular functions (MF), and cellular components (CC).

### 2.7 Latent causal variable analysis

We employed a latent causal variable (LCV) model (34) to ascertain potential causal relationships between COVID-19 and chronic multi-site pain. This model accounts for a genetic correlation between the two traits, which is mediated by a latent variable that exerts a causal effect on each trait. To measure the extent of partial causality, we introduced the concept of genetic causality proportion (GCP), which quantifies the influence of trait 1 on trait 2. The GCP ranges from 0 (indicating no partial genetic causality) to 1 (indicating full genetic causality).

### 2.8 Summary-data-based Mendelian randomization

Summary-data-based Mendelian randomization (35)(SMR) is a method that integrates summary statistics from GWAS and eQTL studies under the MR framework to test for an association between gene expression and a target phenotype. We conducted SMR on brain tissue, which had significant enrichment of chronic pain and COVID-19. The source of eQTL were based on two different reference panels, Genotype-Tissue Project (GTEx) (36) and eQTLGen consortium. Understanding the genetic basis of complex phenotypes and investigating the genetic architecture of blood gene expression are the main objectives of the eQTLGen consortium (37) (https://www.eqtlgen.org/). The heterogeneity in dependent instruments (HEIDI) test was performed to evaluate the existence of linkage in the observed association. Significant shared functional genes between COVID-19 and chronic pain were defined as functional genes which passed the threshold (p<0.05) and HEIDI-outlier test (p > 0.01) in SMR analyses of both traits.

## 3 Results

### 3.1 Genetic overlap and correlation between chronic pain and COVID-19

The SNP heritability on the liability scale for multi-site chronic pain and three forms of COVID-19 were estimate by LDSC. The results showed a SNP heritability of 0.077 for chronic multi-site pain. The genetic correlation between chronic multi-site pain and critical COVID-19 was found to be significantly positive (rg = 0.270, P = 4.39E-10). We also identified the significant positive link between hospitalized COVID-19 (rg = 0.355, P = 2.14E-13) and pain. The genetic correlation between COVID-19 and chronic pain was 0.057, and the P value was 0.047. The most overlap rate between the two shapes is only 0.014 (**Table 1**).

**Table 1:** The source of GWAS and genetic correlation between COVID-19 and chronic multi-site pain using LDSCQ.

Diseases	The detailed information			LDSC			inter
	N_cases	N_control	Ancestry	h2	Rg	P	
Chronic Multi-site pain	387649	0	EUR	0.077			
Hospitalized COVID- 19	32519	2062805	EUR	0.004	0.355	2. 14E- 13	0.012
Critical COVID- 19	13769	1072442	EUR	0.007	0.270	4.39E- 10	0.005
COVID- 19	122616	2475240	EUR	0.002	0.057	0.0477	0.014

h2, heritability; rg, genetic correlation; inter, the ratio of overlap of population.

### 3.2 Cross trait meta-analysis between COVID-19 and chronic multi-site pain

In order to obtain pleiotropic SNPs, we used CPASSOC method and established a threshold of pleiotropic SNPs: P< 5E-8 for meta-analysis and P<1E-3 for the single-trait analysis. In our study, a total of 19 loci were identified between COVID-19 and multi-site chronic pain after annotation in FUMA (**Table 2**). Among these, 9 loci were linked to critical COVID-19, and 8 were related to COVID-19. We identified 3 novel loci (5E-8<P<1E-3 and PCPASSOC<5E-8) between critical COVID-19 and multi-site chronic pain. The most significant novel topSNP shared between them was rs7588831 (P_CPASSOC_=4.15E-09; P_chronic pain_ =1.40E-06; P_COVID-19 = _1.12E-06), which mapped to the HSPD1 and HSPE1 genes. The HSPD1 gene encodes heat shock protein 60 (Hsp60) involved in maintaining protein stability, and the HSPE1 gene codes for heat shock protein 10, which functions as a co-chaperone with Hsp60 (38). The most significant topSNP between COVID-19 and chronic pain was rs13135092, which mapped genes SLC39A8 and BANK1. BANK1, located on chromosome 4, contains genetic variants associated with multiple autoimmune diseases, which is expressed in B cells and serves as a link between different signaling functions within cells (39). There were 2 pleiotropic loci between hospitalized COVID-19 and chronic multi-site pain and no new loci were found between COVID-19/hospitalized COVID-19 and chronic pain.

**Table 2:** The pleiotropic loci between COVID- 19 chronic pain (PCPASSOC < 5× 10-8, 5 × 10-8< single trait P value < 1× 10-3).

Locus	TopSNP CHR	BP		A1	A2	BETA	Multi-site chronic pain		Trait	COVID-19		P	Pcpassoc
							SE	P		BETA	SE		
41	rs117169628	16	89262657	A	G	-0.008	0.003	1. 10E-02	Critical COVID- 19	0.157	0.02	4.36E- 15	3.80E- 16
25	rs12534422	7	75263792	T	C	0.006	0.003	5.50E-03	Critical COVID- 19	0.086	0.015	1.34E-08	4.72E-09
17	rs13135092	4	103198082	G	A	0.033	0.004	7.20E- 15	Critical COVID- 19	0.103	0.025	3.96E-05	3.93E- 13
39	rs34186780	14	104027595	G	T	0.01	0.003	1.30E-04	Critical COVID- 19	0.08	0.016	5.53E-07	1.08E-08
15	rs343312	3	146240757	A	G	-0.017	0.005	2.30E-04	Critical COVID- 19	0.169	0.032	1.01E-07	2. 14E-09
16	rs56203712	4	25342606	G	A	-0.02	0.003	6.40E- 12	Critical COVID- 19	-0.039	0.018	3.53E-02	6.57E- 11
45	rs62098013	18	50863861	A	G	0.017	0.003	4.90E- 11	Critical COVID- 19	-0.031	0.015	4.27E-02	1. 15E- 10
3	rs7528932	1	77949129	T	A	0.01	0.002	2.50E-05	Critical COVID- 19	0.08	0.015	6.56E-08	9.70E- 10
10	rs7588831	2	198508016	A	G	-0.012	0.002	1.40E-06	Critical COVID- 19	-0.071	0.015	1. 12E-06	4. 15E-09
22	rs10992729	9	96181075	T	C	-0.016	0.003	4.70E- 10	COVID- 19	-0.012	0.005	1.30E-02	3.43E-09
32	rs11079993	17	50301552	T	G	0.017	0.003	1.90E- 12	COVID- 19	0.01	0.005	3.50E-02	1.75E- 11
14	rs13135092	4	103198082	G	A	0.033	0.004	7.20E- 15	COVID- 19	0.045	0.009	1.35E-07	4.48E- 13
18	rs142415291	6	34755312	C	A	0.016	0.003	5.00E-08	COVID- 19	0.024	0.009	4.80E-03	1.33E-08
29	rs184781326	14	29459234	G	A	0.011	0.005	4.70E-02	COVID- 19	-0.069	0.012	3.42E-09	1.95E-09
13	rs56203712	4	25342606	G	A	-0.02	0.003	6.40E- 12	COVID- 19	-0.015	0.006	1.02E-02	6.98E- 11
10	rs62263345	3	107252190	G	A	0.021	0.004	3.30E- 10	COVID- 19	0.018	0.007	1.49E-02	4.29E-09
6	rs6721975	2	5832667	T	C	-0.017	0.003	4.90E-09	COVID- 19	-0.019	0.007	7.75E-03	4.33E-08
17	rs13135092	4	103198082	G	A	0.033	0.004	7.20E- 15	Hospitalized COVID- 19	0.09	0.017	7.20E- 15	4.48E- 13
16	rs56203712	4	25342606	G	A	-0.02	0.003	6.40E- 12	Hospitalized COVID- 19	-0.032	0.013	6.40E- 12	6.98E- 11

### 3.3 Colocalization analysis

Two loci among them exhibited a PPH4 over 70% and were identified as pleiotropic loci associated with both critical COVID-19 and multi-site in CPASSOC analysis (**Table 3**). The locus with the most PPH4 value (PPH4 = 99.8%) was situated in an intergenic region on chromosome 4 in COVID-19. The second locus, with a PPH4 of 98.12% in critical COVID-19, was mapped to the BANK1 and SLC39A8 gene, also situated on chromosome 2. The corresponding topSNP of this locus is also the most significant SNP between chronic multi-site pain and COVID-19.

**Table 3:** Results from colocalization analysis for each pleiotropic locus identified from CPASSOC.

TopSNP	Chromosome	Position	A1	A2	COVID-19		Multi-site chronic pain			CPASSOC	PPH3	PPH4
					Trait Beta		P-value	Beta	P-value			
rs13135092	4	103198082	G	A	Critical COVID- 19	0.033	7.2E- 15	0.103	3.96E-05	3.93034E- 13	0.006065378	0.98121232
rs7588831	2	198508016	A	G	Critical COVID- 19	-0.071	1. 11E-06	-0.012	1.40E-06	2.07E- 11	0.080583582	0.899075223
rs13135092	4	103198082	G	A	COVID- 19	0.045	1.35E-07	0.033	7.2E- 15	5.72E-21	0.001647996	0.998219709

### 3.4 MAGMA

**Figure 2:**
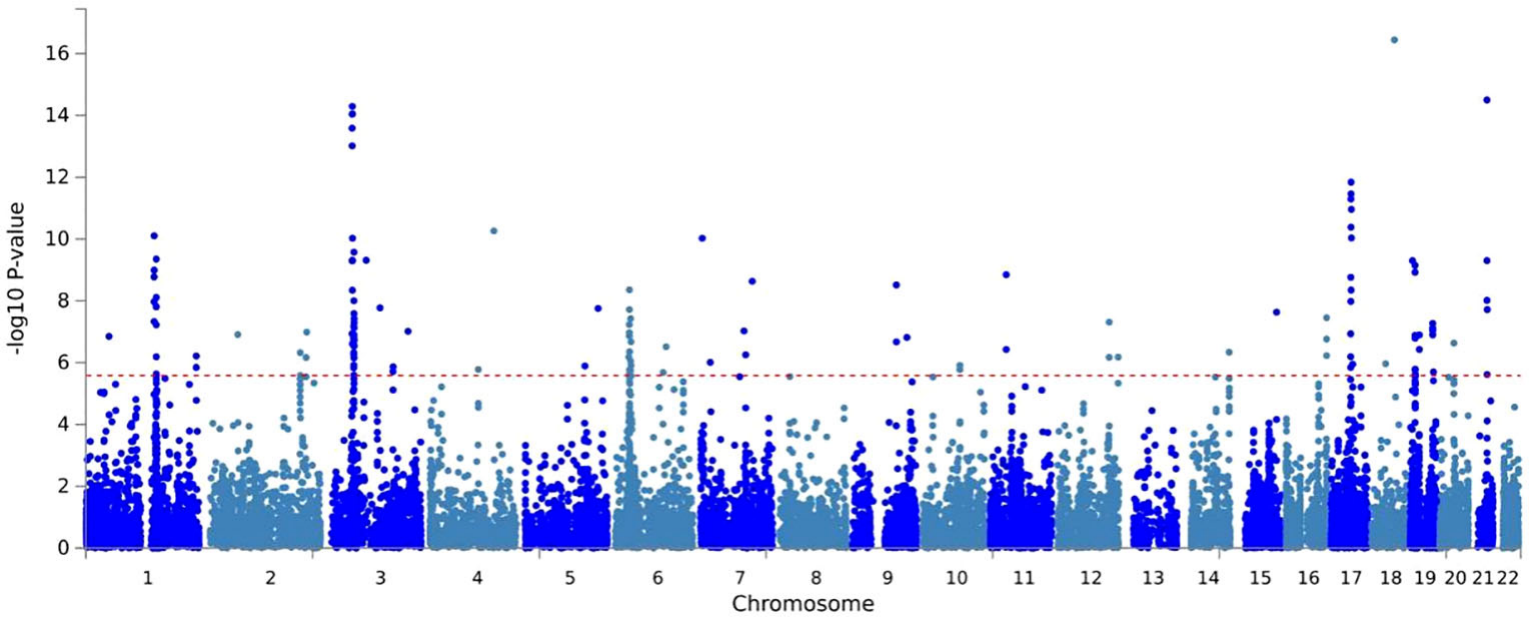
A Manhattan plot of the gene-based test as computed by MAGMA based on results of CPASSOC of critical COVID- 19.

**Figure 3:**
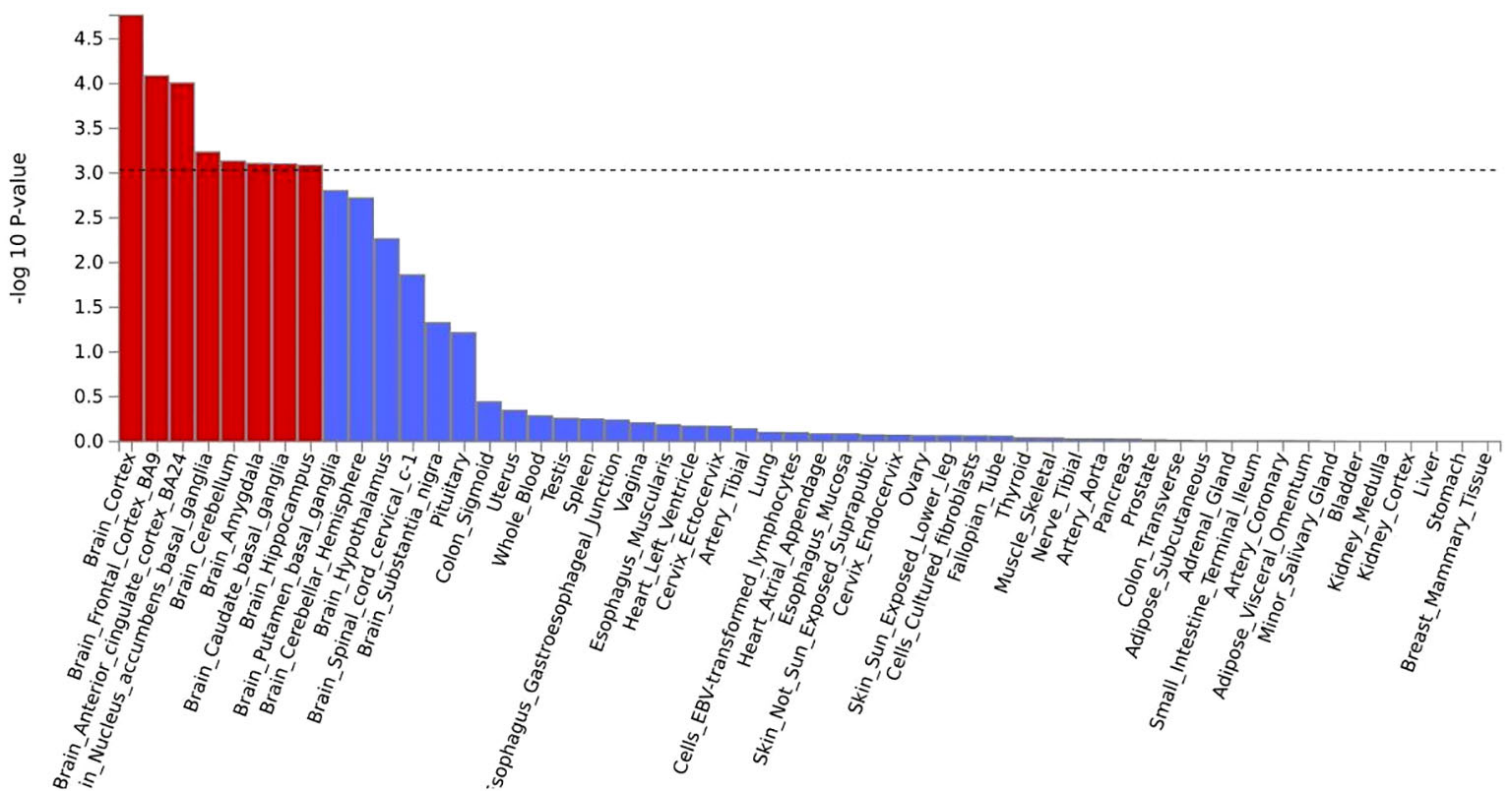
A the result of tissue enrichment in GTEx 8 of critical COVID- 19,the color red represents the significant tissue, the color blue represents the tissue did not reach threshold.

MAGMA analysis was analyzed based on the results of CPASSOC, and we then overlapped genes mapped by FUMA and the result of MAGMA. There were 692 genes annotated in FUMA based on the result of CPASSOC (**Supplementary Table 1**). Among them, there were 251 genes associated with critical COVID-19 (**Figure 2**), 168 genes associated with COVID-19, and 273 genes associated with COVID-19 (**Supplementary Table 2**). It identified 482 significant pleiotropic genes after Bonferroni correction (**Supplementary Table 3**), of which 175 genes were detected in critical COVID-19, and 134 gene were associated with COVID-19. Finally, after correction, we observed significant SNP-heritability enrichment in 12 brain tissues (**Figure 3**), including the brain anterior cingulate cortex BA24, brain cortex, and brain frontal cortex BA9, which exhibited the top three greatest enrichment among the three forms of COVID-19 (**Supplementary Table 4**).

### 3.5 SMR

We selected two forms of COVID-19, critical COVID-19 and COVID-19, for the reason that in this two forms the pleiotropic loci can be detected by COLOC (**Supplementary Table 5**). We totally detected 14 shared genes between critical COVID-19 and chronic pain in the brain cerebellar hemisphere, brain cortex, brain frontal cortex BA9, amygdala, brain hippocampus and other tissues. The gene ANAPC4 was found significantly in the eQTL of lung tissue in both critical COVID-19 (P_SMR_=1.67E-02) and chronic pain (P_SMR_=1.07E-04), the most significant Psmr of COVID-19 is in brain nucleus accumbens basal ganglia (P_COVID-19 = _0.031,P_HEIDI_=0.47) and the corresponding Psmr of chronic pain is 1.46E-05. MARS2 can also found significantly in the tissue of whole blood in both Traits (P_COVID-19 = _1.38E-05,P_chronic pain_=2.20E-05).

### 3.6 Enrichment

**Figure 4:**
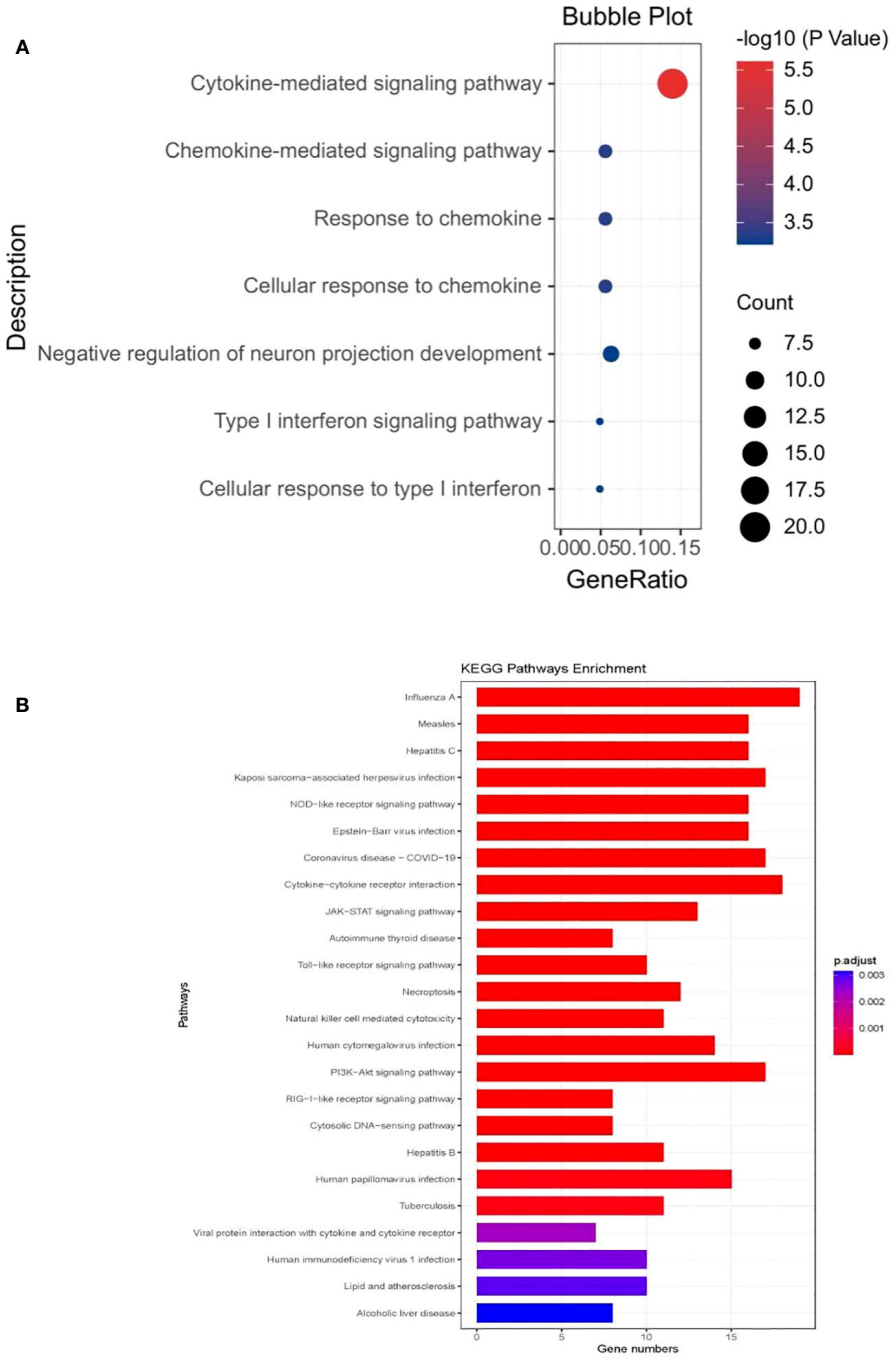
**(A)** The bubble plot of the GO analysis of critical COVID- 19 **(B)** the barplot of KEGG analysis of critical COVID- 19.

The GO analysis revealed that the shared genes between all forms of COVID-19 and chronic pain were significantly enriched in the immune system (**Supplementary Table 6**). In the genes associated with critical COVID-19 (**Figure 4A**), the biological processes were significantly enriched in the cytokine-mediated signaling pathway (P=1.08E-09, Padjust=2.44E-06) and chemokine-mediated signaling pathway (P=4.05E-07,Padjust=4.41E-04). The molecular function was mostly centered around C-C chemokine receptor activity (P=7.44E-12, Padjust=1.85E-09), which was consistent with hospitalized COVID-19 (P=7.05E-12, Padjust=1.74E-09), and COVID-19 (P=9.91E-11, Padjust=1.72E-08). To further understand the functional pathways, KEGG analyses indicated that shared genes were significantly enriched in the pathway of viral protein interaction with cytokine and cytokine receptor in critical COVID-19 (P=3.29E-09, Padjust=9.40E-08).What is more, it is shown that the gene may contribute to NOD-like receptor signaling pathway(P=9.67E-10, Padjust=3.87E-08). We observed that all the shared genes were enriched in COVID-19 (hsa05171) (**Supplementary Table 7**; **Figure 4B**).

### 3.7 LCV

In this analysis, we found the shared pathway (modeled by the LCV) had a large effect on critical COVID-19 but a small effect on chronic pain, Therefore, critical COVID-19 would be highly genetically causal for chronic pain (P=0.15,GCP=0.60). The P values between COVID-19 (P=0.61)/hospitalized COVID-19 (P=0.05) and chronic pain were not significant, which meaned that the correlated pleiotropic relationship may not have causal relationship.

## 4 Discussion

This study was the first comprehensive analysis of the shared genetic basis between COVID-19 and chronic pain using large GWAS. Firstly, we ascertained a significant correlation between COVID-19 and chronic pain. Secondly, our investigation validated 19 pleiotropic loci at the SNP level, indicating their potential impacts on both chronic pain and COVID-19. Thirdly, the LCV analysis provided valuable insights into partial causality between them. Fourthly, we affirmed the correlation between gene expression and both traits, particularly in specific brain tissues.

From a genetic analysis perspective, we conclusively establish that the genes in two loci exhibit pleiotropic effects, which harbored the HSPD1 and HSPE1 genes encoding HSPs. HSPs are proteins produced under cellular stress, activated, and overexpressed to uphold protein structure and function, thereby preventing aggregation, abnormal folding, and degradation. Additionally, they may also be induced by toxins, oxidizing agents, and other stressors. Prior studies have identified HSPD1 mutations as crucial genes in COVID-19 infection, serving as potential drug targets for treating the disease (40). Alreshidi et al. proposed that aspirin-induced HSPs can bind to S-ACE2 and/or AngI, rendering ACE2 less accessible to SARS-CoV-2 and thereby inhibiting cytokine storm (41). Chronic pain formation involves the production of nitric oxide, reactive oxygen species (ROS), and inflammatory cytokines in response to pain stimuli, while the expression of HSPs plays an influential role in the pain reduction by regulating this process (42). HSPs 60 can also suppress Treg cells and downregulate effector T cell migration to ease inflammatory pain (43). Previous findings suggested that the potent anti-inflammatory characteristics of HSPs have therapeutic potentials by enhancing cellular protection to manage pain conditions in neurodegenerative diseases (44). In summary, the decrease of HSPs could be a physiological factor shared by COVID-19 and chronic pain, mutations in inflammation-related genes play a significant role in the susceptibility to both diseases. In the future, targeting heat shock proteins could be developed as a therapeutic approach for managing long-term symptoms of COVID-19.

At the transcriptomic level, we found that ANAPC4 emerged as a shared key gene in both COVID-19 and chronic pain. Anaphase Promoting Complex Subunit 4 (ANAPC4) encodes a protein that constitutes the anaphase promoting complex (APC), a crucial ubiquitin ligase for eukaryotic cell-cycle progression (45). Previous research has established a connection between ubiquitin system malfunction and chronic pain, particularly neuropathic and inflammatory pain (46). In cellular settings, the ubiquitination of the epidermal growth factor receptor (EGFR) can modulate the EGFR/mitogen-activated protein kinase (MAPK) signaling, influencing the outcome of neuropathic pain (47). The involvement of the accumbal ubiquitin-proteasome system is vital in the susceptibility to chronic pain and the development of hedonic behavior (48). On the other hand, the inhibition of glycine has been associated with increased spontaneous activity of spinal cord nociceptive neurons during inflammatory pain, while the activity of the glycine receptor subunit (GlyRs- α 1) is linked with ubiquitination (49). Additionally, our investigation highlights the MARS2 gene as a pivotal factor in both gene and mRNA level analyses. Also known as methionyl-tRNA synthetase 2, MARS2 is responsible for encoding the MARS2 protein, playing a significant catalytic role in the synthesis of methionine transfer RNA within cells. Alterations in methionine metabolism are prevalent metabolomic changes in the chronic pain phenotype (50). Furthermore, methionine-enkephalin has been identified as a regulator of pain signal transmission (51). Previous studies have reported that N-formylmethionine (fMet) contributes to the activation of neutrophils in the context of COVID-19 (52). In summary, the co-occurrence of COVID-19 and chronic pain may depend on alterations in the ubiquitin system and amino acids.

Lastly, we found that tissue enrichment of pleiotropic genes is mostly located in the brain, including regions such as the amygdala and prefrontal cortex, which regulate emotions and stress. COVID-19. During a specific period, it is a stressor that has been associated with the development of long-term symptoms such as insomnia and depression. Numerous studies have demonstrated the significance of the amygdala’s function and anatomical size in this development. Tu et al. discovered that the bilateral hippocampus and amygdala increase in volume, and the increased gray matter volume (GMV) in the left hippocampus and amygdala is negatively correlated with post-traumatic stress disorder (PTSD) scores (53) after recovering from COVID-19. The brain predominantly contains acute pain regulatory areas within its primary somatosensory center (54), while chronic pain encompasses regions associated with cognitive and emotional assessment (55). Increased activity in the Anterior Cingulate Cortex (ACC) can be observed among both healthy individuals and patients with osteoarthritis when utilizing a computerized sustained attention task, Continuous Performance Task (CPT) (56). Chronic pain may lead to alterations in cognitive functions, and these changes may be linked to modifications in the dorsolateral prefrontal cortex (57). Therefore, alterations in brain cognition or structure may serve as an intermediary link in the impact of COVID-19 on pain sensitivity.

Moreover, the enrichment of functions and pathways has revealed that pleiotropic genes can modify signal pathways mediated by cytokines or chemokines, aligning with our hypothesis that genes may contribute to the co-occurrence of diseases through their influence on the immune system. The molecular functions indicated by GO suggested that the genes act as messengers, transmitting signals between cells. C-C chemokine receptors regulate biological processes such as cell migration, proliferation, and inflammation response. C-X-C motif chemokine ligand 10 (CXCL10/IP10) has been widely reported to be elevated in the plasma of COVID-19 (58), and targeting the chemokine ligand 2-chemokine receptor 2 axis provides potential for immunotherapy in chronic pain (59). Finally, utilizing a potential causal model analysis, we identified causal components in the genetic correlation between COVID-19 and chronic pain, consistent with the previous Mendelian randomization study by Zhao (60). The GCP above 60% between critical COVID-19 and chronic pain indicated that the occurrence is largely attributable to the severe illness of COVID-19. However, based on the results of the aforementioned series of studies, it can still be indicated that chronic pain at different stages is genetically correlated with COVID-19.

Certainly,this study does have several limitations that should be acknowledged. First, the sample size of the GWAS data used in this study for COVID-19 was moderate, especially the number of COVID-19 cases, implying that larger-scale GWAS investigations may be more informative in the future. Furthermore, the ethnic community included in this study was predominantly of European heritage, limiting the generalizability of our findings to other ethnicities. Another issue is the lack of validation through laboratory experiments. Lastly, although this study obtained fewer pleiotropic loci, they were selected through a progressively detailed analysis of SNP, gene, and mRNA, enhancing the robustness of the results.

## 5 Conclusion

In conclusion, we reported a genetic overlap between chronic pain and COVID-19. The findings underscore substantial involvement of the inflammatory system, particularly in relation to heat shock proteins. This genetic insight forms a sturdy foundation for enhancing prognosis, preventive strategies, and potentially identifying novel targets for the management and treatment of chronic pain during the COVID-19 period.

## Data availability statement

The datasets presented in this study can be found in online repositories. The names of the repository/repositories and accession number(s) can be found in the article/**Supplementary Material**.

## Author contributions

YC: Conceptualization, Data curation, Formal analysis, Investigation, Methodology, Writing – original draft. PL: Conceptualization, Data curation, Formal analysis, Methodology, Writing – original draft. ZZ: Conceptualization, Data curation, Investigation, Writing – original draft. YY: Conceptualization, Data curation, Investigation, Writing – original draft. SY: Conceptualization, Data curation, Supervision, Writing – original draft. CF: Conceptualization, Data curation, Software, Writing – original draft. WZ: Formal analysis, Project administration, Writing – review & editing. JL: Funding acquisition, Project administration, Writing – review & editing.
