# Corrigendum: Genetic overlap and causality between COVID-19 and multi-site chronic pain: the importance of immunity

**DOI:** 10.3389/fimmu.2024.1419163

**Published:** 2024-04-26

**Authors:** Yanjing Chen, Ping Liu, Zhiyi Zhang, Yingling Ye, Sijie Yi, Chunhua Fan, Wei Zhao, Jun Liu

**Affiliations:** ^1^ Department of Radiology, Second Xiangya Hospital, Central South University, Changsha, China; ^2^ Fujian University of Traditional Chinese Medicine, Fuzhou, China; ^3^ Clinical Research Center for Medical Imaging in Hunan Province, Changsha, Hunan, China

**Keywords:** GWAS, COVID-19, genetic overlap, pleiotropy, chronic multi-site pain, inflammation

In the published article, there was an error in affiliation 1. Instead of “Department of Radiology, Second Xiangya Hospital, South University, Changsha, China”, it should be “Department of Radiology, Second Xiangya Hospital, Central South University, Changsha, China”.

The authors apologize for this error and state that this does not change the scientific conclusions of the article in any way. The original article has been updated.

